# Instantaneous Metabolic Cost of Walking: Joint-Space Dynamic Model with Subject-Specific Heat Rate

**DOI:** 10.1371/journal.pone.0168070

**Published:** 2016-12-28

**Authors:** Dustyn Roberts, Howard Hillstrom, Joo H. Kim

**Affiliations:** 1 Department of Mechanical and Aerospace Engineering, New York University, Brooklyn, New York, United States of America; 2 Leon Root, M.D. Motion Analysis Laboratory, Hospital for Special Surgery, New York, New York, United States of America; Montclair State University, UNITED STATES

## Abstract

A subject-specific model of instantaneous cost of transport (ICOT) is introduced from the joint-space formulation of metabolic energy expenditure using the laws of thermodynamics and the principles of multibody system dynamics. Work and heat are formulated in generalized coordinates as functions of joint kinematic and dynamic variables. Generalized heat rates mapped from muscle energetics are estimated from experimental walking metabolic data for the whole body, including upper-body and bilateral data synchronization. Identified subject-specific energetic parameters—mass, height, (estimated) maximum oxygen uptake, and (estimated) maximum joint torques—are incorporated into the heat rate, as opposed to the traditional *in vitro* and subject-invariant muscle parameters. The total model metabolic energy expenditure values are within 5.7 ± 4.6% error of the measured values with strong (R^2^ > 0.90) inter- and intra-subject correlations. The model reliably predicts the characteristic convexity and magnitudes (0.326–0.348) of the experimental total COT (0.311–0.358) across different subjects and speeds. The ICOT as a function of time provides insights into gait energetic causes and effects (e.g., normalized comparison and sensitivity with respect to walking speed) and phase-specific COT, which are unavailable from conventional metabolic measurements or muscle models. Using the joint-space variables from commonly measured or simulated data, the models enable real-time and phase-specific evaluations of transient or non-periodic general tasks that use a range of (aerobic) energy pathway similar to that of steady-state walking.

## Introduction

Evaluating metabolic energy expenditure (MEE) is critical in a wide range of basic and applied research areas, including gait analysis [1–5], sports science [6,7], engineering design [8,9], fatigue and obesity studies [10,11], and comparative and evolutionary biomechanics [12,13]. In conventional laboratory settings, indirect calorimetry is used to estimate MEE from the rate of oxygen uptake ($\dot{V}O_{2}$) [6,7,14–16]. However, the predictive capabilities of such methods are limited by experimental protocols (e.g., steady state, aerobic metabolic range) and equipment (limited tasks and ranges of motion, time delay between the onset of energy expenditure and when the $\dot{V}O_{2}$ reaches steady state) [7]. For these reasons, only the metabolic measurement of conventional tasks such as steady-state walking [3,4,17] and cycling [7] have been extensively studied, even though steady-state activities are the exception rather than the norm during real-world tasks [18]. In this regard, a recent study introduced a dynamic systems approach that estimates instantaneous metabolic rate during non-steady-state walking from measured data [18]. However, even in those conventional tasks, the results in general are not always reliable (e.g., disagreements involving the relative metabolic costs of gait stance and swing phases [1]). Also, since even a simple task is not performed with identical kinematics and dynamics by a subject on different trials, which result in different MEE, the use of empirically constructed formulas [7,16] or look-up tables can produce significant errors [19].

The MEE models scaled from experimental energetics of individual muscle activations [13,20,21] avoid the use of $\dot{V}O_{2}$ measurement and the associated limitations. The instantaneous evaluation from models enables integration over a phase-specific time duration to estimate the relative metabolic costs of different phases of a task [1]. However, there are two main problems in current muscle energy models: redundancy and unknown interactions. Multiple musculotendon units that cross a given joint can be activated as synergists to generate a resultant moment of muscle forces about the joint center [22]. Due to the large number and redundancies of functional skeletal muscles in a human body, the excessive number of degrees of freedom (DOFs) makes deterministic and verifiable evaluation difficult. In addition, due to the compliance in the geometric and mechanical configurations of soft tissue, the muscle space is essentially infinite dimensional. Although this redundancy is often addressed through optimization to predict a unique muscle activation pattern [23], most models still struggle when attempting to predict submaximal activation patterns [24]. As a result, existing muscle energy models may demonstrate conflicting results, especially in the case of eccentric (lengthening) contractions [25].

Furthermore, the energetics of the human musculoskeletal system is not simply a scaled version of the energetics of a single muscle commonly modeled using line segment, because interactions between muscles and their surroundings (other muscles, bones, and adipose tissue) are often difficult to measure or predict. These time-varying interactions, including normal and frictional contacts, wrapping and sliding with the internal surroundings [26], change of moment-arms [26], and compliance due to elastic tissues [24,27], alter the transmission of muscle forces to their resultant joint actuator torque, which become more severe when the joint angles deviate far from neutral positions. These complexities and indeterminacies indicate that the evaluation of MEE from muscle-space energy models (usually obtained from *in vitro* measurements) does not consistently provide reliable results. For instance, even in normal walking, the predicted MEE results from existing muscle-space models often include significant errors (up to 50%) [25,28,29]. Furthermore, the quantitative models of molecular-level muscle energetics in the current literature are mostly hypothetical and not validated [30,31]. Consequently, the validity and applications of existing muscle energy models are limited to specific conditions, tasks, and certain muscle types (e.g., skeletal muscles that can be approximated as straight lines during a given task).

A plausible solution to the aforementioned problems is to model the MEE in joint space. The resultant effect of multiple muscles that contribute to the rotation of a single anatomical joint can be mapped to a combination of one or more kinematically equivalent revolute joints [26,32]. The relative angles of these revolute joints form the generalized coordinates of joint space. Under a rigid-body assumption for each segment, joint space is finite dimensional, from which the whole-body segmental configurations can be uniquely determined using independent DOFs. Unlike those in muscle space, the joint-space kinematic and kinetic variables are readily available from commonly measured or simulated movement data. Furthermore, the maximum-torque-angle-velocity relationships in joint space [33,34] are simpler than the maximum-force-length-velocity relationships in muscle space [24,27]. For these reasons, joint-space formulations are commonly used in inverse dynamics for motion analysis [26,35–37] and computational simulation and optimization [38–40]. Several recent studies have demonstrated that MEE during walking can be predicted using joint-space kinematic and kinetic data [41,42]. However, due to the lack of an accurate MEE model in joint space, incomplete proxies for MEE, such as center-of-mass work [43,44], segmental work [43,45], modified total mechanical work [45], rate of normalized absolute joint moment impulses [43], mechanical work derived from experimental efficiencies [46,47], and other combinations [48], are often used as approximations in the literature.

In this study, instantaneous evaluation of energetic cost of transport (COT) is introduced from the derivation of a novel subject-specific model of MEE rate in joint space. The COT is a quantity (formulated either without [49,50] or with dimensions [51]) that provides a measure of energy economy and is defined as the total energy expended during locomotion per unit distance and body weight or mass. While the energy efficiency of human gait is formulated inconsistently without unique definition throughout existing studies [36,46,47,52,53], the inverse of COT quantifies the “locomotion efficiency” and allows for non-dimensional comparisons across different subjects and gait strategies [49–51]. However, the common total COT values are lumped over a time duration due to the aforementioned limitations of metabolic measurement. The instantaneous COT (ICOT) is defined in this study (Section 2.4) as the COT evaluated at each time instant, rather than for a finite time interval. The ICOT can provide a deeper analysis and insights about the energetic causes and effects between the body and gait parameters as a function of time or gait cycle. (Note: This study introduces the first use of the acronym ICOT defined as the “instantaneous cost of transport”. The definition of iCOT in a previous study [54] seems to be a typo and is inconsistent with other work, e.g., [55], from the same authors in which iCOT is correctly defined as incremental cost of transport.) Unlike the previous joint-space model that was estimated from empirical formula [42], the new models are established through refined model identifications, subject-specific parameters, and metabolic measurements. The MEE, ICOT, and phase-specific COT models are first derived theoretically by combining the laws of thermodynamics and the principles of multibody system dynamics. Distinct from a few inductive, empirical muscle energy models that were also described with respect to the first law of thermodynamics [25,56–58], the proposed deductive approach includes rigorous mathematical formulations and systematic identifications of energy transformation (among various energy components) and transfer (through external versus internal work and heat). The generalized heat rates as functions of subject-specific system parameters in joint space, which are mapped from muscle energetic properties, are estimated from walking data. The experimental walking data with different speeds and metabolic measurements used for estimation and validation are processed for the whole-body energetics, which includes upper-body and bilateral data synchronization.

## Methods: Modeling and Experiments

The main focus of this work is to identify and derive physically accurate terms and forms in the MEE model. The MEE and ICOT models as functions of joint kinematic and dynamic variables are mathematically derived. Then the subject-specific heat rates in joint space are estimated from experimental whole-body walking data.

### Model Constraints, Identification, and Derivation

The entire human body is identified as the system of interest for thermodynamic analysis in order to include the metabolic energy component (internal biochemical energy) explicitly as one of the energy terms, which would not be possible if only muscles were selected. In the proposed derivations, the changes of system energy due to mass transfer (e.g., air, sweat, and food) and chemical reactions for metabolism (between air and biochemical energy) at the system boundary are not taken into account. While the inhaled or exhaled air through respiration is not included in the system, it is assumed that sufficient amount of metabolic energy source is readily available within the human body throughout the duration of a given task. This is a plausible constraint with respect to the scope of this study, which encompasses the transfer and transformation between metabolic energy, mechanical work, heat, and other various chemically-nonreactive energy components. Furthermore, the derivations from the first law are not dependent on either how or at what rate the energy source is produced from metabolism, or the internal process of energy transfer and transformation within the human body. Whether the energy transformation includes chemical reaction or not is also one of the criteria for the following breakdown of system energy into components. Therefore, the derived model terms are valid for tasks that utilize metabolic pathways both with (i.e., aerobic) and without (i.e., anaerobic) oxygen, which is only important as an oxidizing agent, as long as there is no instantaneous shortage (or fatigue) in the available metabolic energy source. The differences due to aerobic versus anaerobic pathways can be reflected in the model through the heat coefficient parameter values that are experimentally identified (the walking experiments in this study are for aerobic conditions only). In addition, while the heat dissipation due to muscle actuation is a significant portion in the models, additional heat exchange due to sweat is assumed to be negligible. These model constraints imposed on the energetic effects of respiration and sweat allow closed-system thermodynamic analysis, and are plausible assumptions for natural tasks like normal walking for a brief period of time after an initial period of rest.

The energy components associated with the human multibody dynamic system include the kinetic (${\dot{E}}^{k}$), external potential (${\dot{E}}^{p}$; corresponds to the work done by all external conservative forces), internal potential or strain (${\dot{U}}^{p}$; corresponds to the work done by all internal conservative forces), internal thermal (${\dot{U}}^{t}$), and metabolic ($-{\dot{E}}^{met}$; where ${\dot{E}}^{met}$ is the MEE rate) energy rates, where upper dots indicate time-derivatives. Another term (${\dot{E}}^{o}$) includes various energy components that are not directly related to muscle activities, for instance, those at the tissue level for the brain, kidneys, liver, etc. (which have no muscle at all) and those for the molecules transportation through the GI tract membranes. The rate of change in the total energy is incorporated into the first law for a closed system:

$${\dot{E}}^{k}+{\dot{E}}^{p}+{\dot{U}}^{p}+{\dot{U}}^{t}+{\dot{E}}^{o}-{\dot{E}}^{met}={\dot{W}}^{ext}+{\dot{Q}}^{ext}$$ (1)

where ${\dot{W}}^{ext}$ is the work rate done by non-conservative external forces and ${\dot{Q}}^{ext}$ is the heat transfer rate across the system boundary. The segment volumes (pressure-volume work) and contact properties (elasticity, viscosity, and friction) of the system elements are assumed to be independent of the heat and thermal energy. The MEE rate can be derived in terms of the rate of work ${\dot{W}}^{int}$ done by non-conservative internal forces by incorporating the work-energy principle [59] of dynamics (${\dot{E}}^{k}+{\dot{E}}^{p}+{\dot{U}}^{p}={\dot{W}}^{int}+{\dot{W}}^{ext}$) into the above equation:

$${\dot{E}}^{met}={\dot{W}}^{int}+{\dot{U}}^{t}-{\dot{Q}}^{ext}+{\dot{E}}^{o}$$ (2)

The heat rate $\left({\dot{U}}^{t}-{\dot{Q}}^{ext}\right)$ is due to the net effects of increased internal thermal energy and outbound heat transfer. In general, the heat and internal work result from actuation and dissipation within the system, where the actuation is generated by the activations of skeletal, smooth, and cardiac muscles.

### Generalized Coordinates and Heat Rates: Muscle to Joint Space Mapping

**Fig 1:**
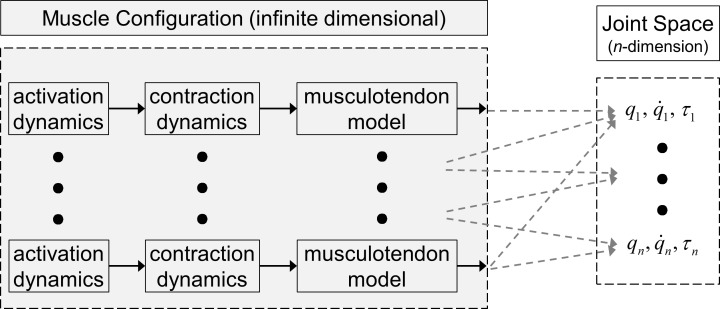
Mapping from muscle space to joint space

The angles between adjacent link segments of the human body serve as the generalized coordinates in joint space. If the human body configuration can be represented using *n* revolute joints (*n* DOFs) in addition to the position and orientation of a body base with respect to an inertial frame, the joint space is composed of vectors of *n* independent generalized coordinates *q*_*i*_, *i* = 1, …, *n*. The kinematic constraints imposed on the musculotendon systems by anatomical structure result in one effective DOF angle for each revolute joint that is dependent on the muscle and musculotendon lengths. As a result, each joint angle (and velocity as its total time-derivative) is a function of the associated muscle and musculotendon lengths (and contraction velocities), representing the mapping from the infinite-dimensional muscle space to the finite-dimensional joint space, in which the generalized coordinates (joint angles) are uniquely determined (Fig 1).

At each joint, the muscle-induced actuator torque *τ*(*t*) is the resultant of the moments of the associated muscle forces about the joint axis of rotation. Since each muscle force’s orientation and position of its point of application (including the varying moment-arm length and shape) with respect to the rotational axis depend on their contact and wrapping with the surroundings [26], each joint actuator torque is a function of the associated muscle forces and musculotendon lengths. In this study, to avoid the complexities and indeterminacies in multiple musculotendon dynamics, the joint actuator torque serves as a generalized torque for the corresponding generalized coordinate (joint angle) (Fig 1). This mapping incorporates the activation, contraction, and musculotendon dynamics and their maximum-force-length-velocity properties [24,27] into the actuator torque dynamics at each joint along with the maximum-torque-angle-velocity properties [33,34]. The remainders of the agonist and antagonist muscle forces that correspond with moment equilibrium contribute to active stiffness as cocontraction, which can only be measured using EMG, and is indeterminate and not uniquely identifiable in joint space. In this approach, the cocontraction at a joint is divided into torque-dependent (by active joint stiffness required for basic structural and motor control stability) and torque-independent (due to excessive muscle activations that are arbitrary and not essential for a given activity) components [42]. While torque-dependent cocontraction is the minimum requirement for a given task for a subject, the torque-independent cocontraction is unnecessary surplus.

The heat that active skeletal muscles dissipate depends on their voluntary activations. Similar to the generalized coordinates and torques, the activations of the associated muscles at each revolute joint constitute the generalized activation in joint space [42], which can be derived as a function of *τ*(*t*) using the common Hill-type muscle contraction dynamics [24,27,60]. The generalized activation is used along with the skeletal muscle heat-activation characteristics [20,21,24,61,62] to derive generalized heat rates in joint space [42]. The generalized activation-maintenance heat rate is:

$${\dot{Q}}_{i}^{am}(t)=h_{i}^{am}|\tau_{i}(t)|+h_{i}^{am\varepsilon }+h_{i}^{am1}(t)\;(i=1,\ldots ,n)$$ (3)

where $h_{i}^{am}$ is the coefficient associated with the joint actuator torque and torque-dependent cocontraction, $h_{i}^{am\varepsilon }$ is the coefficient associated with basal torque-dependent cocontraction, which represents the minimal activations by skeletal muscles required for structural integrity and motor control stability even when *τ*(*t*) is zero, and $h_{i}^{am1}$ is the time-varying coefficient associated with the active stiffness due to torque-independent cocontraction. Both $h_{i}^{am}$ and $h_{i}^{am\varepsilon }$ are functions of the joint angle, velocity ($\dot{q}$), and maximum (across angles and velocities for the joint) actuator torque (*τ*^*max*^), incorporating muscle’s maximum-force-length-velocity properties. The generalized shortening-lengthening heat rate is:

$${\dot{Q}}_{i}^{sl}(t)=h_{i}^{sl}|\tau_{i}(t){\dot{q}}_{i}(t)|+h_{i}^{sl\varepsilon }+h_{i}^{sl1}(t)|\tau_{i}(t)|+h_{i}^{sl2}(t)|{\dot{q}}_{i}(t)|+h_{i}^{sl3}(t)\;(i=1,\ldots ,n)$$ (4)

where $h_{i}^{sl1}$, $h_{i}^{sl2}$, and $h_{i}^{sl3}$ are the time-varying coefficients associated with the active stiffness due to torque-independent cocontraction. The coefficients $h_{i}^{sl}$ and $h_{i}^{sl\varepsilon }$ are associated with the joint mechanical power and basal torque-dependent cocontraction, respectively, and both are functions of the joint angle, velocity, and maximum actuator torque incorporating muscle’s maximum-force-length-velocity properties.

The above terms corresponding to torque-independent cocontraction (the last in ${\dot{Q}}_{i}^{am}$ and the last three in ${\dot{Q}}_{i}^{sl}$) can be considered to be voluntarily arbitrary. Therefore, these arbitrary terms can be re-grouped as an independent variable, the generalized torque-independent cocontraction heat rate ${\dot{Q}}_{i}^{cc}(t)$ at each DOF. Likewise, the terms corresponding to the basal torque-dependent cocontraction can be re-grouped as a single variable ${\dot{Q}}_{i}^{\varepsilon }$:

$${\dot{Q}}_{i}^{cc}(t)=h_{i}^{am1}(t)+h_{i}^{sl1}(t)|\tau_{i}(t)|+h_{i}^{sl2}(t)|{\dot{q}}_{i}(t)|+h_{i}^{sl3}(t)\;\text{and}\;{\dot{Q}}_{i}^{\varepsilon }=h_{i}^{am\varepsilon }+h_{i}^{sl\varepsilon }\;(i=1,\ldots ,n)$$ (5)

In this approach, the number of variables is reduced significantly, which is another advantage of joint-space formulation.

### Joint-Space Dynamic Models of MEE Rate and ICOT

From the definition of generalized torques, the total work rate done by skeletal muscle forces is equal to the total joint work rate ${\dot{W}}^{joint}=\sum_{i=1}^{n}\tau_{i}(t){\dot{q}}_{i}(t)$ done by joint actuator torques (i.e., net mechanical power summed over all joints). Therefore, the total internal work is the sum of the work done by the joint actuator torques, smooth muscles (${\dot{W}}^{smooth}$), and cardiac muscles (${\dot{W}}^{cardiac}$):

$${\dot{W}}^{int}={\dot{W}}^{joint}+{\dot{W}}^{smooth}+{\dot{W}}^{cardiac}=\sum_{i=1}^{n}\tau_{i}(t){\dot{q}}_{i}(t)+{\dot{W}}^{smooth}+{\dot{W}}^{cardiac}$$ (6)

The total heat rate is the sum of the generalized heat rates ${\dot{Q}}_{}^{am}$ and ${\dot{Q}}_{}^{sl}$ from skeletal muscle activation and the heat from the activations of smooth muscles (${\dot{Q}}^{smooth}$) and cardiac muscles (${\dot{Q}}^{cardiac}$). The fractions of the skeletal muscle heat that correspond to torque-independent and basal torque-dependent cocontraction in the above derivations can be split into separate terms:

$$\begin{array}{l} {\dot{U}}^{t}-{\dot{Q}}^{ext}={\dot{Q}}_{}^{am}+{\dot{Q}}_{}^{sl}+{\dot{Q}}^{smooth}+{\dot{Q}}^{cardiac}\\ \;=\sum_{i=1}^{n}h_{i}^{am}\left|\tau_{i}(t)\right|+\sum_{i=1}^{n}h_{i}^{sl}\left|\tau_{i}(t){\dot{q}}_{i}(t)\right|+\sum_{i=1}^{n}{\dot{Q}}_{i}^{cc}(t)+\sum_{i=1}^{n}{\dot{Q}}_{i}^{\varepsilon }+{\dot{Q}}^{smooth}+{\dot{Q}}^{cardiac} \end{array}$$ (7)

The heat and work terms due to involuntary activations (for vital organs and structures) of smooth and cardiac muscles, the heat of the basal torque-dependent cocontraction (which can be assumed to be relatively small and invariant for a given subject from a mechanical perspective) by skeletal muscle activations, and the other energy component due to tissue-level sources [63] can be combined to constitute the basal metabolic rate (BMR), ${\dot{E}}^{bmr}$:

$${\dot{E}}^{bmr}={\dot{W}}^{smooth}+{\dot{W}}^{cardiac}+{\dot{Q}}^{smooth}+{\dot{Q}}^{cardiac}+\sum_{i=1}^{n}{\dot{Q}}_{i}^{\varepsilon }+{\dot{E}}^{o}$$ (8)

The BMR is the minimal energy expenditure at rest without visible body movement. In general, the BMR may vary within and across the trials even for a given subject. However, in this study, possible variation of the BMR is assumed to be negligible, which is supported by the experimental protocol. Thus, despite the complexity involved in decomposing the BMR [63], the BMR is regarded as one of the subject-specific system parameters in the models.

Rearranging the above equations, the total MEE rate (in watts) as a function of time is:

$${\dot{E}}^{met}(t)={\dot{W}}^{int}+{\dot{U}}^{t}-{\dot{Q}}^{ext}+{\dot{E}}^{o}=\sum_{i=1}^{n}\tau_{i}(t){\dot{q}}_{i}(t)+\sum_{i=1}^{n}h_{i}^{am}\left|\tau_{i}(t)\right|+\sum_{i=1}^{n}h_{i}^{sl}\left|\tau_{i}(t){\dot{q}}_{i}(t)\right|+\sum_{i=1}^{n}{\dot{Q}}_{i}^{cc}(t)+{\dot{E}}^{bmr}$$ (9)

This equation represents the dynamic model of the MEE rate in terms of state variables (${\dot{q}}_{i}$), control inputs (*τ*_*i*_ and ${\dot{Q}}_{i}^{cc}$), and system parameters ($h_{i}^{am}$, $h_{i}^{sl}$, and ${\dot{E}}^{bmr}$). The time-varying state variables and control inputs depend on the specific motion’s kinematics and dynamics, and thus can incorporate any transient or steady-state motion. Note that, the joint velocities and actuator torques in this model, as well as in experimentally measured inverse-dynamics data (below), are the results of the activation-contraction-musculotendon dynamics of the associated muscles. According to the second law of thermodynamics, any heat transfer to the body cannot be stored or recycled as metabolic energy. Therefore, the components in the heat terms are always non-negative, where the absolute values reflect the contributions of both positive and negative variables.

In contrast to the first law that is stated in terms of external work and heat [64], the MEE rate is formulated explicitly in terms of internal work and heat, which depend only on the relative internal quantities, i.e., joint velocities and actuator torques. In particular, the relative quantities in the joint work formulation do not require the use of a specific reference frame as in the calculation of the center-of-mass work or total work that are commonly used in the literature (e.g., [65,66]; these other forms of work cannot be used for the first law derivations). This allows the consistent use of generalized coordinates (joint angles) and the associated quantities (velocities and torques) in each term and provides additional advantages of joint-space formulations. In addition, the internal work term inherently includes the contributions of soft-tissue work (${\dot{U}}^{p}$) that is known to be non-negligible in some human tasks like walking [65], which would otherwise be needed as an explicit term.

The dimensionless COT at a time instant (ICOT) and each gait phase (phase-specific COT) can then be calculated as follows:

$$ICOT(t)=\frac{{\dot{E}}^{met}(t)}{Mgv(t)}$$ (10)

$$COT=\frac{\frac{1}{T}\int_{T}{\dot{E}}^{met}(t)dt}{Mg\left(\frac{1}{T}\int_{T}v(t)dt\right)}$$ (11)

where *M* is body mass, *g* is the gravitational acceleration, and *v*(*t*) is the instantaneous speed of the body’s center of mass. These models can be evaluated for any time interval of interest *T*, not only for an entire gait cycle (*T*_*total*_), but also for its constituent gait phases (*T*_*SS*_ for single support (SS) and *T*_*DS*_ for double support (DS) phases, respectively) and even at a given time instant. Both ICOT and phase-specific COT are based on ${\dot{E}}^{met}(t)$, which includes the BMR, and are different from locomotion COT, which excludes the BMR, or mechanical COT, which is based on positive mechanical work only [67]. While the structures of the models are mathematically derived, determining the forms and parameter values of the heat coefficient functions ($h_{i}^{am}$ and $h_{i}^{sl}$) requires estimation based on experiments. Note that these generalized heat coefficient functions in joint space reflect the effects of the contributing muscles’ energetic properties.

### Experiments and Data Processing

Ten healthy subjects (Table 1) provided written informed consent according to a protocol approved by the Hospital for Special Surgery Institutional Review Board. In addition to height, mass, and age, the maximum isometric extension torque ($\tau_{knee}^{max}$) at the right knee was measured for each subject with a Biodex dynamometer (Biodex Medical Systems) to estimate the maximum joint torques at all DOFs. The knee joint was positioned at a flexion angle of 60°, which allows production of the theoretical maximum isometric knee extension torque as determined through existing data [34] and OpenSim software.

**Table 1:** Descriptive statistics for all subjects used in the estimation (mean ± standard deviation) and validation of the models^a^.

Group	Subjects	Sex	Height (m)	Mass (kg)	Age (yrs)	Max Torque (Nm)
Estimation	Subjects 1,3,4,6	M	1.83 ± 0.06	87.9 ± 10.9	31 ± 6	212.4 ± 66.2
	Subjects 2,5,8,10	F	1.62 ± 0.07	57.1 ± 10.0	32 ± 9	107.6 ± 35.7
Validation	Subject 6	M	1.79	68.5	21	284.4
	Subject 7	M	1.63	52.6	20	208.4
	Subject 8	F	1.75	75.0	32	124.1
	Subject 9	F	1.56	52.2	23	109.7

^a^Data from Subjects 6 and 8 are included in both the estimation and validation groups. Maximum torque values are for the right knee.

**Fig 2:**
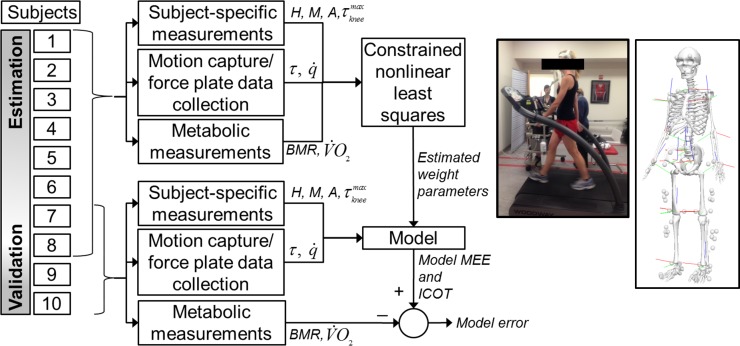
Data processing flow chart (left; the subject numbers shown are different from the actual experiments for schematic illustration), metabolic testing on treadmill (inset), and whole-body model with marker sets and local coordinate frames (right)

The experimental data were divided into two groups: estimation and validation (Table 1 and Fig 2). All of the trials from six subjects and the majority of the trials from two other subjects were included in the estimation group. These trials were used to estimate the heat coefficient functions, and the resulting complete models were evaluated from the validation data. The validation data included 2–3 stride cycles (defined here as a right heel contact to the next right heel contact) at each walking speed from two subjects not represented in the estimation group (Subjects 7 and 9), and one stride at each walking speed from two subjects (Subjects 6 and 8) also included in the estimation group. Student’s t-tests were used to confirm there were no statistical differences in mass, height, age, or maximum knee torque between the estimation and validation groups.

#### Metabolic Measurements Using Indirect Calorimetry

A metabolic cart (TrueOne® 2400 Metabolic Measurement System, Parvo-Medic, Inc.) was used to measure oxygen uptake $\dot{V}O_{2}$ (liters O_2_/minute) and carbon dioxide output $\dot{V}CO_{2}$ (liters CO_2_/minute). Initially, the BMR was obtained during quiet sitting over a six-minute interval. The average of the last three minutes was determined as the BMR. Although standing BMR is sometimes used to determine net MEE in gait analysis, sitting BMR was used in this study to separate the MEE required to maintain a standing posture, which is attributed to the skeletal muscle heat terms.

Each subject was asked to walk for 3 to 5 minutes on a treadmill (Woodway, Inc., WI) and choose a preferred walking speed with self-selected stride frequency and length. Then the experimental protocol of five test speeds was started using this preferred walking speed (100%), followed by 70%, 85%, 115%, and 130% of the preferred walking speed. The full protocol consisted of 25 total minutes (five minutes for each speed) of treadmill walking with rest periods of two minutes between trials. The short duration of each trial and the sufficient resting time between the trials ensure the effects of fatigue, sweat loss, and possible variation of the BMR were minimized. Subjects were instructed to relax during the trials to avoid unnecessary (i.e., torque-independent) cocontraction. Average $\dot{V}O_{2}$ was recorded for each minute during the five-minute period at each speed, and the average of the last three of these measurements was determined as the representative $\dot{V}O_{2}$ for a given speed with units converted to watts [14]. Note that this unit conversion method is equivalent to that described by Brockway [68] as long as the respiratory exchange ratio is less than unity, which signifies that aerobic metabolism is the main metabolic pathway [5]. To confirm the validity of the conversion, the respiratory exchange ratio was monitored during the experiment at each speed [53,69].

#### Upper-Body and Bilateral Data Synchronization for Whole-Body Kinematics and Kinetics

Since the metabolic testing provided the MEE for the whole body, it was necessary to process the whole-body kinematics and kinetics, so that associations could be made between the data sets. A whole-body reflective marker set (a standard 6-DOF set for lower body [70] and a Rab marker set [71] for upper body) was used to define the head, upper arm, forearm, hand, torso, pelvis, thigh, shank, and foot segments of each subject (Fig 2). This provided a 42-DOF (*n* = 42) three-dimensional human model consisting of 3 DOFs for each of the 2 ankles, 2 knees, 2 hips, 2 wrists, 2 elbows, 2 shoulders, 1 neck, and 1 waist. Each segment was defined by a local coordinate system according to the International Society of Biomechanics standards [72,73]. After standing static calibration trials, kinematic and kinetic data were collected as each subject walked over ground at the same five walking speeds as during the metabolic testing. Each trial data were matched with the respective metabolic data collected for each test speed, which is commonly supported by the equivalency between over-ground and treadmill walking [74]. Kinematic data were collected at 120 Hz using a twelve-camera motion capture system (Motion Analysis Corporation, CA). Ground reaction forces were measured concurrently at 4800 Hz using four force plates (two Bertec Corp, OH, and two AMTI, MA). Subject speed was monitored using a pair of photogates straddling the 30-m walkway and only trials that were within 5% of the target speed were retained. Steady-speed walking was verbally encouraged during each trial, and confirmed through observation when processing motion capture data.

The coordinates for the reflective markers and the ground reaction force data were obtained using Cortex software (Motion Analysis Corporation) and was exported for further processing in Visual3D software (C-Motion Inc.). Joint torques were then calculated using inverse dynamics and inertial parameters for each segment identified by the marker sets [75,76].

Gait event detection was implemented and all joint variables and torques per stride along with other descriptive statistics (e.g., stride length) were exported to text files for subsequent processing in MATLAB (The MathWorks, Inc.). To get whole-body data, the upper body and the right leg data were exported for each right leg stride captured. Then the left leg data were exported for each left leg stride captured. A whole-body stride was created by dividing the left stride data at the right heel strike event, then combining it with the upper body and right leg data such that at any given time instant the left leg data were synchronized with the corresponding right leg time instant.

### Subject-Specific Parameters and Constrained Nonlinear Least-Squares Estimation of Heat Coefficient Functions

In general, the MEE varies across different subjects even with identical kinematic and kinetic input variables. To account for the different energetic properties across subjects (and DOFs), relevant subject-specific parameters are identified and incorporated into the heat coefficient functions. The system parameters associated with energetic properties were identified by extensive least-squares error analyses among several measurable parameters, and were confirmed using correlation analyses [77,78] for all subjects. The strong correlations between the experimental average MEE rates and each of the measured parameters, including mass (*M*), height (*H*), maximum rate of oxygen uptake ($\dot{V}O_{2}^{max}$), and maximum knee torque ($\tau_{knee}^{max}$), were identified at the subject’s preferred walking speeds (see Results section). Measuring $\dot{V}O_{2}^{max}$ that reflects the aerobic fitness of an individual involves an intensive fatiguing test protocol, which is accompanied with potential risk and would have been impractical for this study. Alternatively, it can be estimated from the ratio between maximum and resting heart rate [79], where the maximum heart rate can be estimated from age [80]. In this initial study, age (*A*) was used as a substitute for $\dot{V}O_{2}^{max}$ in the heat coefficient functions. In addition, from the above derivations, the generalized heat coefficient functions $h_{i}^{am}$ and $h_{i}^{am}$ at each DOF depend on the respective maximum actuator torque. The maximum actuator torque at *i*th DOF is estimated by linearly scaling the measured $\tau_{knee}^{max}$ with ${\tilde{\tau }}_{i}^{max}/{\tilde{\tau }}_{knee}^{max}$, where ${\tilde{\tau }}_{i}^{max}$ and ${\tilde{\tau }}_{knee}^{max}$ are the average human’s maximum torque of *i*th DOF and knee, respectively, from available data (e.g., [34,81,82]). In this way, $\tau_{knee}^{max}/{\tilde{\tau }}_{knee}^{max}$ serves as a subject-specific ratio that scales the DOF-specific ${\tilde{\tau }}_{i}^{max}$. This linear scaling provides a relative measure of each subject’s strength at each DOF (DOF-specific) as well as across different subjects (subject-specific). Using these subject-specific system parameters as independent variables, the heat coefficient functions at each DOF (assumed right-left symmetric) are formulated as a product of two terms—divided according to their dependencies on DOF—using the method of separating variables:

$$h_{i}^{am}=(w_{0}^{am}+w_{1}^{am}M+w_{2}^{am}A+w_{3}^{am}H)\left(1+w_{4}^{am}\frac{\tau_{knee}^{max}}{{\tilde{\tau }}_{knee}^{max}}{\tilde{\tau }}_{i}^{max}\right)\;(i=1,\ldots ,n)$$ (12)

$$h_{i}^{sl}=(w_{0}^{sl}+w_{1}^{sl}M+w_{2}^{sl}A+w_{3}^{sl}H)\left(1+w_{4}^{sl}\frac{\tau_{knee}^{max}}{{\tilde{\tau }}_{knee}^{max}}{\tilde{\tau }}_{i}^{max}\right)\;(i=1,\ldots ,n)$$ (13)

where $w=[w_{0}^{am},w_{1}^{am},w_{2}^{am},w_{3}^{am},w_{4}^{am},w_{0}^{sl},w_{1}^{sl},w_{2}^{sl},w_{3}^{sl},w_{4}^{sl}]$ are the weight parameters that will be solved for through the constrained nonlinear least squares algorithm, and each term in the product is approximated as a linear polynomial function of the associated variables (linear regression). Note that the second terms in these products are both subject- and DOF-specific.

To estimate the unknown weight parameters **w** in the heat coefficient functions, the differences between the model MEE values and the experimental MEE values are minimized as a constrained nonlinear least squares problem (Fig 2). Since only the time-averaged total amount of MEE (${\bar{E}}^{met}$) can be obtained from experimental measurements, the proposed model MEE rate is integrated over the total time duration (*T* = *T*_*total*_) for *N* number of strides and implemented into the average residual. The following (squared) root-mean-square error is used as the cost function:

$$error(w)=\frac{1}{2}\frac{{\left\|\left[{\left({\bar{E}}^{met}-\int_{T}{\dot{E}}^{met}(t)dt\right)}_{k}\right]\right\|}^{2}}{N}\;(k=1,\ldots ,N)$$ (14)

The irreversibility of the MEE and heat dissipation based on the second law of thermodynamics is imposed through inequality constraints. The non-negativity of heat dissipation implies $h_{i}^{am}\geq 0$ and $h_{i}^{sl}\geq 0$ (*i* = 1, …, *n*), and thus each subject- and DOF-based term must satisfy the following inequalities:

$$w_{0}^{am}+w_{1}^{am}M+w_{2}^{am}A+w_{3}^{am}H\geq 0;\;1+w_{4}^{am}(\tau_{knee}^{max}/{\tilde{\tau }}_{knee}^{max}){\tilde{\tau }}_{i}^{max}\geq 0\;(i=1,\ldots ,n)$$ (15)

$$w_{0}^{sl}+w_{1}^{sl}M+w_{2}^{sl}A+w_{3}^{sl}H\geq 0;\;1+w_{4}^{sl}(\tau_{knee}^{max}/{\tilde{\tau }}_{knee}^{max}){\tilde{\tau }}_{i}^{max}\geq 0\;(i=1,\ldots ,n)$$ (16)

In addition, since the expended metabolic energy cannot be recharged by negative joint work, the net MEE (excluding the BMR and cocontraction term) at each DOF should be nonnegative at all times:

$$\tau_{i}(t){\dot{q}}_{i}(t)+h_{i}^{am}\left|\tau_{i}(t)\right|+h_{i}^{sl}\left|\tau_{i}(t){\dot{q}}_{i}(t)\right|\geq 0\;(i=1,\ldots ,n\;\text{for}\;\forall t)$$ (17)

While the torque-independent cocontraction at each DOF is arbitrarily controlled, its heat dissipation is always non-negative. Since the total MEE on average is bounded from above by the subject-specific maximum oxygen uptake ($\dot{V}O_{2}^{max}$ in watts), the torque-independent cocontraction heat for a given time duration *T* is also bounded from above accordingly:

$$0\leq \int_{T}{\dot{E}}^{met}(t)dt\leq \dot{V}O_{2}^{max}\cdot T\;\text{and}\;{\dot{Q}}_{i}^{cc}(t)\geq 0\;(i=1,\ldots ,n\;\text{for}\;\forall t)$$ (18)

Since subjects were instructed to relax and walk naturally during all trials in this study, it is assumed that torque-independent cocontraction heat is negligible as compared with other model terms, and is not included in the least squares estimation. Note, on the other hand, that the torque-dependent cocontraction that exists in natural walking is included in the coefficient functions $h_{i}^{am}$, $h_{i}^{sl}$ and the BMR ${\dot{E}}^{bmr}$.

The algorithm is implemented with the *fmincon* subroutine in MATLAB. The resulting weight parameters, which are used to complete the models, are independent of motion or subject, and thus their estimated values from these steady-state normal walking can be used in the evaluation of any subject’s general tasks (time-varying state variables and control inputs) that are transient or non-periodic.

## Results

The values of the average (± standard deviation) preferred walking speed (1.29 ± 0.11 m/s) and the corresponding stride length (1.48 ± 0.11 m) for all subjects’ trials are within normal ranges for healthy adults [46]. The walking data show typical characteristic patterns [46] for the two input variables of the models, i.e., joint velocities and torques. The respiratory exchange ratio was monitored and remained less than unity in all trials for all subjects, which validates the conversion approach used to calculate the MEE in watts.

**Fig 3:**
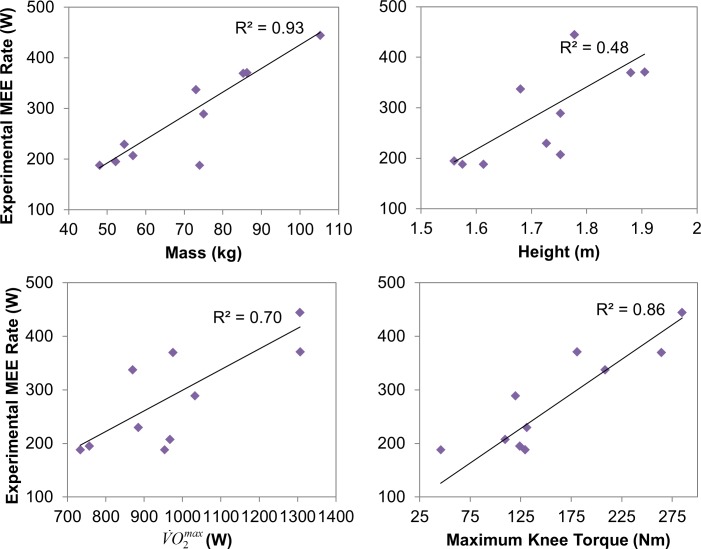
Correlation plots of experimental MEE rate at preferred walking speed versus subject-specific parameters for all subjects

The inclusion of mass, height, $\dot{V}O_{2}^{max}$ (estimated from age), and maximum joint torques (estimated from $\tau_{knee}^{max}$) in the heat coefficient functions is supported by correlation analyses at each subject’s preferred walking speed (Fig 3), which were guided by similar correlation analyses in the literature [78]. No significant difference in mass (*p* = 0.95), height (*p* = 0.69), $\dot{V}O_{2}^{max}$ (*p* = 0.89), or maximum knee torque (*p* = 0.67) was found between the estimation and validation groups.

**Fig 4:**
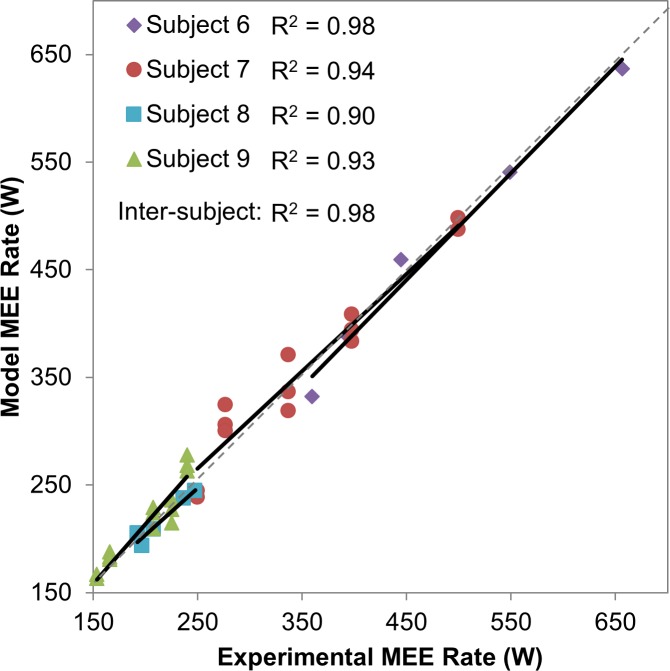
Inter- and intra-subject correlations between time-averaged model and experimental MEE rates for all trials in the validation group. Dashed line indicates ideal slope for zero errors.

The unknown weight parameters **w** in the joint-space heat coefficient functions were determined from the constrained nonlinear least squares algorithm using the estimation group experimental data (Table 2). To account for the different dimensions and scales across the subject parameters, the values shown in Table 2 were obtained by normalizing the subject parameters by their respective maximum order of magnitudes (100 for mass, age, and maximum joint torques and 10 for height). The results were then incorporated into the model and used to evaluate the models from the validation group. The mean of the time-averaged model MEE rates for the validation group was within 5.7 ± 4.6% absolute error of the experimental indirect calorimetry values. Strong inter-subject (R^2^ = 0.98) and intra-subject (R^2^ = 0.90–0.98) correlations were noted (Fig 4).

**Table 2:** Results of the constrained nonlinear least squares algorithm for weight parameters estimation

$w_{0}^{am}=-2.27\times 10^{-4}$	$w_{1}^{am}=-6.40\times 10^{-5}$	$w_{2}^{am}=1.61\times 10^{-4}$	$w_{3}^{am}=1.43\times 10^{-3}$	$w_{4}^{am}=1.82\times 10^{3}$
$w_{0}^{sl}=9.79\times 10^{-1}$	$w_{1}^{sl}=3.45\times 10^{-2}$	$w_{2}^{sl}=1.07\times 10^{0}$	$w_{3}^{sl}=-1.46\times 10^{0}$	$w_{4}^{sl}=-6.97\times 10^{-3}$

**Fig 5:**
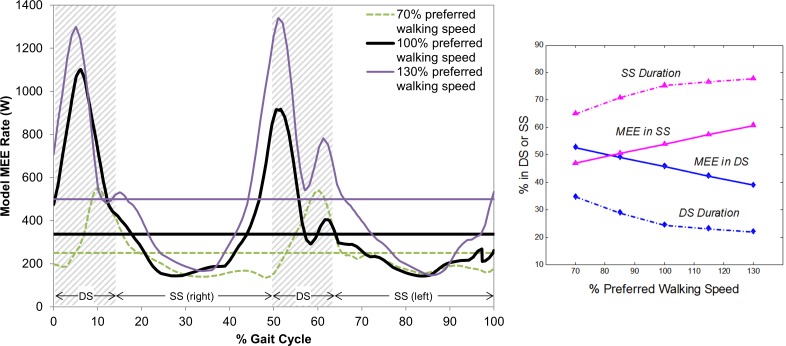
Instantaneous model MEE rates for 70%, 100%, and 130% preferred walking speed of Subject 7 (from the validation group) as a function of percentage gait cycle (left). Horizontal lines indicate experimental mean MEE rates for the respective walking speeds. Percent of gait cycle duration (from experiments) and MEE (from the model) in SS and DS on average across all validation subjects (from each subject’s averages) at different speed conditions (right).

The instantaneous MEE rates at different walking speeds are also predicted as a function of percent gait cycle (Fig 5 - left). Integrating the instantaneous MEE rates over phase-specific time intervals *T*_*SS*_ and *T*_*DS*_ provides the average MEE rates during SS and DS phases, respectively. The percent of gait cycle duration and MEE for SS increase as the walking speed increases, while those for DS decrease (Fig 5 - right). On average across all the validation subjects’ average strides, the two DS phases account for a decreasing portion of the gait cycle duration and the model MEE per gait cycle, dropping from 34.9 to 22.1% and from 52.8 to 39.2%, respectively, as walking speed increases. The respective remainders correspond to the increases in the percent gait cycle duration and MEE in SS phases per gait cycle as the walking speed increases.

**Fig 6:**
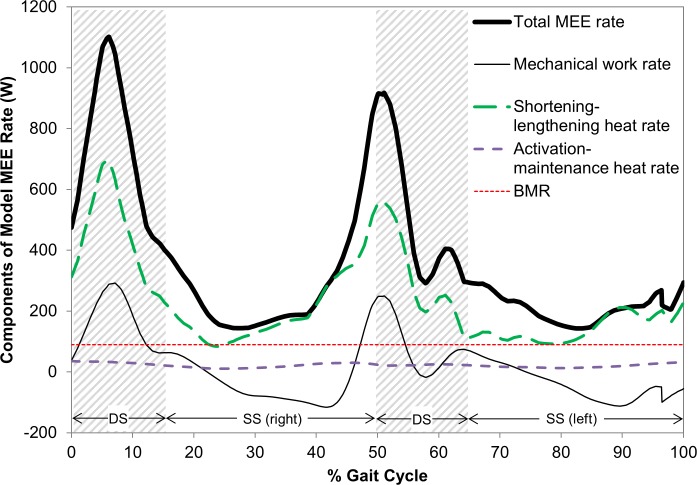
Components of model MEE rate for one stride at the preferred walking speed of Subject 7

The individual contributions of the main terms—joint work, generalized heat, and BMR—in the MEE rate as well as those of the generalized heat components (${\dot{Q}}_{}^{am}$ and ${\dot{Q}}_{}^{sl}$) can also be predicted from the model. The result from a preferred walking speed stride of a validation subject is illustrated as an example (Fig 6).

**Fig 7:**
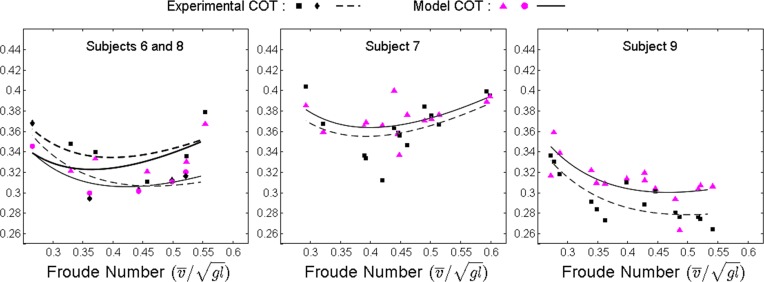
Model and experimental total COT versus dimensionless walking speed for all validation trials of each subject

For each subject, the total (*T* = *T*_*total*_) model and experimental COT versus speed curve can be approximated from all validation trials using a basic Laurent polynomial form (Fig 7). The dimensionless walking speed represented using the Froude number $\bar{v}/\sqrt{gl}$, where $\bar{v}$ is the average walking speed and *l* is leg length (estimated as 0.53 multiplied by the height [46]), is used to normalize the speed across the subjects of a broad range of dimensions (Table 1) [83–85].

**Fig 8:**
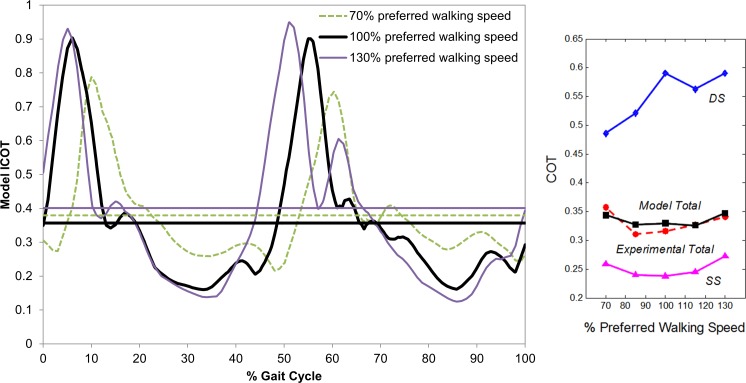
ICOT (from the model) for 70%, 100%, and 130% preferred walking speed of Subject 7 (from the validation group) as a function of percentage gait cycle (left). Horizontal lines indicate experimental total COT values for the respective walking speeds. Model COT values in SS, DS, and total gait cycle and experimental total COT values on average across all validation subjects (from each subject’s averages) at different speed conditions (right).

The ICOT model results are illustrated as a function of percent gait cycle for different walking speeds (Fig 8 - left). As the walking speed increases, the peaks in the ICOT appear earlier in the gait cycle and the ranges between the maximum (peak) and minimum ICOT values are larger. While the total COT can be obtained from both the experiments and the model, these instantaneous evaluations are available only through the proposed approach. In addition, the instantaneous MEE model enables the calculation of not only the total gait cycle COT, but also its breakdown (i.e., phase-specific) into SS and DS COT (Fig 8 - right) by integrating over *T*_*SS*_ and *T*_*DS*_, respectively, which are not available from conventional experiments. The model total COT values (0.326–0.348) averaged across all validation subjects (using each subject’s averages) reliably predicted the magnitude and the convexity along walking speed of those from experiments (0.311–0.358). While the DS COT curve shows roughly increasing but irregular patterns, the SS COT demonstrates a clear convex curve with respect to walking speed. Overall, the DS COT (0.486–0.591) and SS COT (0.239–0.274) values are consistently larger and smaller, respectively, than those of total gait cycle.

## Discussion

This study introduces the dynamic models of MEE and ICOT in joint space with subject-specific energetic properties. The generalized heat coefficient functions that complete the models were formulated as functions of the measurable subject parameters—mass, height, age (as an indirect implementation of $\dot{V}O_{2}^{max}$), and maximum joint torques (also DOF-specific)—which was qualified from strong correlations between each of these parameters and experimental time-averaged MEE rates (Fig 3), and were approximated from weight parameters estimation (Table 2). The positive weight parameter values for the age term in the heat coefficient functions also imply increased COT (due to increased torque-dependent cocontraction) in older adults.

### Subject-Specific MEE Rate Model in Joint Space: Accuracy and Validity

The main focus of this work is the derivation of physically accurate terms and forms in the MEE model, rather than the prediction of accurate numeric values. Nevertheless, the resulting heat coefficient functions led to a versatile MEE model capable of accurate predictions (within 5.7 ± 4.6% average absolute error of the measured values) of the total MEE across subjects and speeds. The inter-subject and intra-subject correlations between the model and experimental MEE values of all trials in the validation group are also very strong (Fig 4). This demonstrates the ability of the proposed model to predict reliable MEE values consistently across different subjects and different ranges of walking speeds within the same subject. Possible sources of errors include the approximations used in the derivations and inaccuracies in the experimental values. Although the respiratory exchange ratio was less than unity during all trials (which validates the conversion approach), these errors may be due to the assumptions inherent in the indirect calorimetry measurement or conversion method [14], or inaccuracies in the measurement equipment (e.g., treadmill calibration). Nevertheless, in comparison with the existing muscle energy models that may overestimate the MEE by up to 50% [1,28,29], the errors from the proposed models are relatively and consistently low. The improved accuracy in the proposed model is likely due to the incorporation of the subject-specific parameters in the estimation of the joint-space heat coefficient functions directly from active human data, as opposed to the traditional *in vitro* and subject-invariant parameters used by most muscle energy models. Note that the accuracy achieved through the proposed model is not solely due to the common relationship between walking speeds and time-averaged metabolic rates. The derived distribution of the heat coefficient functions to the body joints enables the non-uniform contribution of each joint to the total MEE. In particular, the DOF-specific term in the heat coefficient function $h_{i}^{am}$ implies that, for a given subject, stronger joints (with larger maximum actuator torque or maximum strength) have larger heat coefficient values, and thus contribute to larger portions of MEE in generating the same actuator torque. For instance, the MEE at the hip will be larger than that at the ankle when generating the same joint actuator torque or mechanical power, due to the difference in the maximum actuator torque values (e.g., 240.1 N.m for hip vs. 151.5 N.m for ankle used in this study for male subjects). Despite the relatively low heat coefficient values in the sagittal plane at the ankle as compared to the hip, the ankle’s joint actuator torque and mechanical power during walking result in its larger MEE, which may be in line with its larger active muscle volume [12].

The instantaneous model MEE rates (and ICOT, as discussed below) as functions of time or percent gait cycle (Fig 5) and their breakdown into the various energetic components (Fig 6) can only be validated indirectly (from various perspectives), because similar data from experiments that would allow direct comparison does not exist in the current literature. In addition to the aforementioned mean model MEE rates that show strong correlations (Fig 4) with the respective experimental values, the curve patterns are similar among the subjects with periodic attributes. As in the experimental mean MEE rates, the instantaneous MEE rate at a given percentage of the gait cycle increased as speed increased. Also, the peaks in the MEE rate curves in all trials correspond with the DS phases. Although the percentage DS phase durations decrease from 34.9 to 22.1% of the gait cycle time as speed increases, which is consistent with a previous experimental study [85] (the major results in this reference for pediatric gait characteristics are described to be consistent with those of adults), the model shows that they are responsible for the decrease from 52.8 to 39.2% of the total MEE per gait cycle (Fig 5). This trend of the MEE rate curves and their similarity with those of the joint mechanical power (Fig 6) are also in line with previous studies showing that the mechanical work during step-to-step transition that occurs in parallel with the DS phase is a major determinant of the metabolic cost of walking [1,17,86,87]. It is interesting that, at the preferred walking speed, the average % MEE for DS in the current study is about 46% (Fig 5), which falls between the previously reported ~70% [87] and 37% [1,86] for step-to-step transitions. The peaks of the joint mechanical power occurring during the DS phases, in which more muscles are engaged to support the body, explain the higher metabolic demand at these phases due to the increase in muscular activities. Those for the SS phases demonstrated the opposite patterns. The percent durations of SS per gait cycle were constantly larger than those of DS for all speed conditions. However, the percent MEE values of SS were smaller than those of DS for slow walking speed, and became larger as the speed increased. Overall, for both the SS and DS phases, the increasing/decreasing trends of the average percent MEE rates along walking speed were consistent with those of the respective percent durations per gait cycle. Additional aspects of the validity are discussed below along with those of the COT and ICOT.

### Total COT: Model versus Experiments

The total COT plots of each subject (versus normalized speed; Fig 7) and all validation subjects’ averages (versus percent of preferred walking speed; Fig 8) demonstrate the characteristic convexity (as approximated by a Laurent polynomial form) in both the experimental and model curves that are consistent with the results in existing literature [4,43,51,88]. These curves have a minimum at or near the preferred walking speed of each subject and subjects’ averages, respectively, and are relatively flat for a wide range of speeds surrounding the preferred walking speed. This is also consistent with the results in an extensive experimental study [47], in which the experimental COT showed a notable increase only at very high or very low speeds of walking. Also, the ranges of the model total COT for each subject curve (0.26–0.41) and for all validation subjects’ averages (0.326–0.348) agree with the current experimental values (0.311–0.358) and encompass the average experimental value of 0.3 at preferred walking speeds reported in the literature [50], [67]. These results demonstrate that the subject-specific joint-space models established in this study are able to predict the trend and magnitudes in total COT observed at different walking speeds in different subjects.

### ICOT and Phase-Specific COT

Since the ICOT is effectively the MEE rate normalized by subject weight and velocity at a given time instant, the ICOT as a function of time or percent gait cycle (Fig 8) follows a similar periodic pattern as in the MEE rates (Fig 5). Like MEE rates, the maximum peaks of the ICOT curves occur during DS phases, and are shifted down and forward in time as speed decreases, which is in agreement with joint kinetics during walking found in an experimental study [85]. The range between the maximum and minimum ICOT values increases as the speed increases. On the other hand, there are notable differences between the instantaneous MEE rate and ICOT. The peaks and average of ICOT profiles are less sensitive to walking speeds as compared with those of MEE rates. The ratio of the maximum peak values of the ICOT for 70%, 100%, and 130% preferred walking speed of a subject is 0.82:1.00:1.05, while that of the MEE rate is 0.59:1.00:1.46. The ratio of the mean values of the ICOT, 1.02:1.00:1.05, is even smaller than that of the MEE rate, 0.74:1.00:1.48, of the same subject’s trials. This low sensitivity of the ICOT with respect to instantaneous walking speed is due to the nature of its quantification. While the MEE rate increases with walking speed for a given subject at a time instant, the ICOT is calculated from the division of the increased MEE rate by the increased instantaneous speed. This point becomes clearer by comparing the minimum values of the MEE rates and ICOT both occurring during SS phases; while the minimum MEE rates are similar across different speeds (0.97:1.00:1.16 for 70%, 100%, and 130% preferred walking speed), the ICOT results illustrate noticeably decreased minimum values as speed increases (1.60:1.00:0.87). Generally, the ICOT provides normalized comparison of the energetic costs across different speeds and subjects, which is not available through MEE rate alone. This is in line with the results of the total COT (Fig 7 and Fig 8), in which the minimum occurred near the preferred walking speed, in contrast to the minimum experimental mean MEE rate that occurred at the lowest walking speed (Fig 5).

The results per gait cycle phase illustrate that the DS COT values (0.486–0.591) were consistently larger than those of the total gait cycle and SS phase, and roughly increased as speed increased (Fig 8). On the other hand, the SS COT demonstrated more notable and interesting features that were consistent among all subjects. Like the total COT, the SS COT resulted in a convex curve with its minimum near the preferred walking speed. This is distinct from the percent MEE in SS (Fig 5), which increased as speed increased. The similarity in patterns of the SS COT and the total COT is likely due to the consistently larger percent duration of the SS phases per gait cycle (about 1.9–3.5 times) than those of DS, since the COT calculation depends on the duration of interest. For the same reason, the magnitudes of the SS COT (0.239–0.274), which were constantly smaller than those of the total gait cycle, are closer than those of the DS to the total COT curve. This characteristic is different from that of the percent MEE, in which the ratio of the magnitude of SS relative to DS increased with speed, and the percent MEE values of SS were larger than those of DS for all speed conditions except for the slowest (70% preferred walking speed). In other words, in terms of both the patterns and magnitudes of the COT, the SS phase is more influential than the DS phase.

As another validation aspect, the consistently smaller COT in SS than that in DS is also in agreement with the trade-off between efficiency (as the inverse of COT) and stability of normal human walking [50] in the sagittal plane, where the SS phases are relatively unstable with respect to static [37] and dynamic balance [89]. The contrasting features of the efficiency-stability compromise in the relatively unstable SS versus the relatively stable DS were not available through the percent MEE rates (Fig 5) alone, but through (the inverse of) the phase-specific COT. Note that, while the proposed model can evaluate the ICOT for any unnatural/abnormal gait, these attributes do not always characterize the walking with unnatural compensation due to, for instance, heavy load [90] or disability.

### Concluding Remarks and Future Work

The ICOT and phase-specific COT demonstrated normalized comparison across different speeds and subjects, major influence from the SS phase, and relatively low sensitivity with respect to walking speed, which were not available through the analyses from the instantaneous MEE rates or the total COT (as detailed in Section 4.3). While the model parameters were estimated using normal walking data in this study, there is no inherent limitation on these models for real-time calculations of instantaneous MEE rate and ICOT as functions of time in transient as well as steady-state evaluations of any subject’s general tasks that use a range of energy pathways similar to that of (i.e., aerobic) steady-state walking. For instance, when experimentally measured movement data (available from common gait analysis) are provided as inputs, the models will predict the MEE and ICOT as outputs without $\dot{V}O_{2}$ measurement for complex non-periodic tasks that may not be experimentally verifiable. When experimental data are not provided, the proposed models can be used to predict or computationally simulate movement data as well as the MEE and ICOT in various what-if scenarios. On the other hand, note that, while the model and its parameters are validated against normal walking in this study, the accuracies of the model parameters are not tested for other activities.

Future work will address some of the aforementioned assumptions used in establishing the models. First, the initial experimental data limited validation to steady-state walking. Although walking was chosen due to the wealth of comparable literature, less than 1% of walking actually happens in steady state, a condition required for traditional metabolic measurements [91]. Also note that, although the derived model terms and forms are general and are valid for both aerobic and anaerobic tasks, the parameter values that were estimated through indirect calorimetry in this study are reliable only for aerobic tasks. Future work should extend the model and its experimental validation to running, non-steady-state gait [18], and other general tasks, including those that are aperiodic, submaximal, or have a substantial anaerobic component [92]. Second, open-system thermodynamic analysis, including chemically reacting components, will incorporate the energetic effects of respiration-induced metabolism process (between air enthalpy and biochemical energy), which will allow modeling of the instantaneous change in available metabolic energy source and fatigue. In this regard, incorporating the molecular-level models of muscle energetics [30] may also advance the understanding of the energetic differences among positive, zero, and negative work. Third, for more accurate calculation of the MEE components, inverse dynamics methods should be advanced such that the resultant joint torque can be accurately partitioned into muscle-induced actuator torque and passive reaction moments [93,94]. Finally, future studies will include direct measurements of $\dot{V}O_{2}^{max}$, age-related trend of cocontractions (through EMG), and percent body fat to be considered explicitly as subject-specific parameters.

## Supporting Information

S1 File Matlab Codes Package and Sample Data Files. (ZIP)

S2 File Matlab Package User Manual. (PDF)
