# Supplementary material for: Instantaneous Metabolic Cost of Walking: Joint-Space Dynamic Model with Subject-Specific Heat Rate
Source: PLoS One. 2016 Dec 28;11(12):e0168070. doi: 10.1371/journal.pone.0168070 (PMC5193358; doi:10.1371/journal.pone.0168070)
Supplement: S2 File — (PDF) [file pone.0168070.s002.pdf]

USER MANUAL
Supplemental material to accompany:
D. Roberts, H. Hillstrom, J. H. Kim, "Instantaneous Metabolic Cost of Walking: Joint-Space Dynamic Model with Subject-Specific Heat Rate"

OVERVIEW

The provided folder includes everything needed to run code described in the above-mentioned manuscript, including MATLAB code and Excel spreadsheets. Sample experimental data from five trials/strides with one subject are included. These data were collected as described in the manuscript and processed with Visual3D.[1] The "resultsComprehensive_HSS.xlsx" file contains the subject's basic parameters and "01_Metabolic_test.xlsx" contains the subject's data collected during metabolic testing. The kinematic and dynamic data for each trial/stride are stored in two spreadsheets (to store left and right side data separately) with names such as "70walk_Left1.xlsx" and "70walk_Right1.xlsx". The numerical prefix "70," "85," "115," or "130" indicates the trial walking speed in terms of percent preferred walking speed (the lack of a numerical prefix indicates preferred walking speed). Torque is in Newton-meters and angular velocity is in degrees/second.

Sign conventions follow ISB and other standards [1]–[3]:

Lower body sign conventions

- Hip flexion(+)/extension(-)
- Hip abduction(+)/adduction(-)
- Hip internal rotation (+)/external rotation(-)
- Knee flexion(-)/extension(+)
- Knee abduction(+)/adduction(-)
- Knee internal rotation (+)/external rotation(-)
- Ankle dorsiflexion(+)/plantarflexion(-)
- Ankle abduction(+)/adduction(-)
- Ankle inversion(+)/eversion(-)

Upper body sign conventions

- Neck flexion(+)/extension(-)
- Neck abduction(+)/adduction(-)
- Neck internal rotation (+)/external rotation(-)
- Shoulder flexion(+)/extension(-)
- Shoulder abduction(+)/adduction(-)
- Shoulder internal rotation (+)/external rotation(-)
- Elbow flexion(+)/extension(-)
- Elbow abduction(+)/adduction(-)
- Elbow internal rotation (+)/external rotation(-)
- Wrist flexion(+)/extension(-)
- Wrist abduction(+)/adduction(-)

[1] Custom data files can be used with this code as long as separate files are created for left and right strides at the same speed. A trial/stride is defined from heel strike to heel strike.

- Wrist internal rotation (+)/external rotation(-)
- Waist flexion(+)/extension(-)
- Waist abduction(+)/adduction(-)
- Waist internal rotation (+)/external rotation(-)

TO RUN THE SOFTWARE

1. Unzip the MEE-v2-Portable folder and save in C:\MEE-v2-Portable.
2. Open humanMenu.m and run it to generate model results. It will output the results to an Excel spreadsheet named “Model Results.xlsx”.

TO VIEW THE RESULTS

1. After running humanMenu.m, an output file named “Model Results.xlsx” is created in the MEE-v2-Portable folder. Open the output file to view the main model results. The filename will have a suffix containing the date and time in the following format: “Model Results_20150503_222021.xlsx”. This file contains most of the model results, excluding the instantaneous model results.
2. If you wish to see the instantaneous MEE and the component breakdown of instantaneous MEE rate for subject number *i* and trial/stride number *j*, you will need to run MEEplotter.m by typing

   MEEplotter(*i*,*j*)

   into the command line in MATLAB. In order to run MEEplotter.m you must have already run humanMenu.m prior. MEEplotter will plot the results over a gait cycle output the results to an Excel spreadsheet named “Instantaneous Model Results.xlsx”. The filename will also have a suffix containing the date and time in the form “Instantaneous Model Results_20150503_222021.xlsx”. A sample plot produced by MEEplotter is shown below:

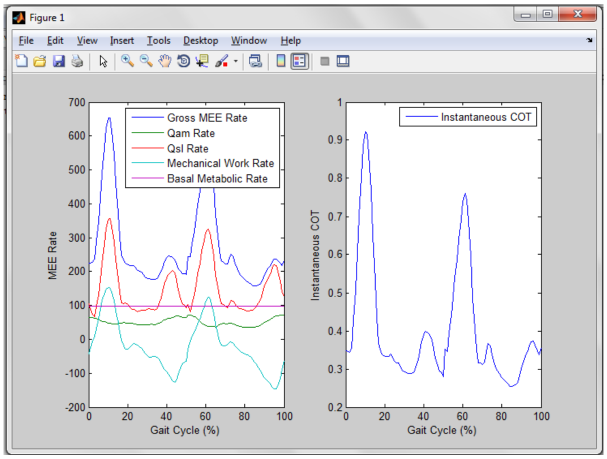

3. Note that all of the experimental data, model results, and more are stored in the structure array named “subject” (accessible in the MATLAB workspace) and that the results are organized in this structure array according to the subject number and trial/stride number.

If you wish to access a value from within MATLAB, simply enter the value's name at the command line in MATLAB in the following format:

subject(*i*).stride(*j*).E_model

where *i* and *j* are the subject number and stride/trial number. For subject specific values such as the following:

subject(i).gender

only the subject number *i* is required.

SUBJECT SPECIFIC VARIABLES STORED IN "SUBJECT" STRUCTURE ARRAY

subject(i).gender	subject's gender
subject(i).mass	subject's mass
subject(i).height	subject's height
subject(i).age	subject's age
subject(i).kneeTorqueMax	subject's maximum knee torque
subject(i).Brate	subject's basal metabolic rate (BMR)

STRIDE/TRIAL SPECIFIC VARIABLES STORED IN "SUBJECT" STRUCTURE ARRAY

subject(i).stride(j).speedR	Average speed of trial
subject(i).stride(j).timeElapsed	Time elapsed during trial
subject(i).stride(j).strideLength	Stride length of trial
subject(i).stride(j).B	Energy consumed by basal metabolism during trial
subject(i).stride(j).DS_percent	Percent of trial in double support phase
subject(i).stride(j).E_Model	Gross MEE (Joules) used during trial
subject(i).stride(j).Enet_Model	Net MEE (Joules) used during trial
subject(i).stride(j).Erate_Model	Average Gross MEE rate (Watts)
subject(i).stride(j).EnetRate_Model	Average Net MEE rate (Watts)
subject(i).stride(j).Qam_Model	Activation-Maintenance Heat (Joules)
subject(i).stride(j).Qsl_Model	Shortening-Lengthening Heat (Joules)
subject(i).stride(j).workSum	Total mechanical work done (Joules)
subject(i).stride(j).E_Model_101	Instantaneous gross MEE rate (Watts)
subject(i).stride(j).Enet_Model_101	Instantaneous net MEE rate (Watts)
subject(i).stride(j).Qam_Model_101	Instantaneous A-M Heat rate (Watts)
subject(i).stride(j).Qsl_Model_101	Instantaneous S-L Heat rate (Watts)
subject(i).stride(j).MEEinDS_Model	MEE consumed while in DS phase
subject(i).stride(j).MEEinSS_Model	MEE consumed while in SS phase
subject(i).stride(j).MEEinDSRatio_Model	Percent MEE consumed while in DS phase
subject(i).stride(j).DSCOT_Model	Cost of transport calculated with the MEE in DS
subject(i).stride(j).SSCOT_Model	Cost of transport calculated with the MEE in SS
subject(i).stride(j).COT_Model	Cost of transport calculated with the total MEE
subject(i).stride(j).iCOT_Model	Instantaneous cost of transport

NOTES

The suffix _Model is used to indicate model results. In most cases, variables with the same name but with an _HSS instead refer to the experimental results obtained with metabolic testing.

The additional suffix _101 indicates that the variable is a time series of length 101 containing the instantaneous model results sampled at 0% to 100% gait cycle and not a single value.

CODE MAP

Nested levels indicate which custom functions are called from where. Boldface text indicates that the function is nested within its parent function.

```
humanMenu
    loadData
        B_HSS
        E_HSS
        E_ACSM
        processSubject
        processStride
            interpGaitCycle
            diffshc
    calcResults
        MEE
            DOFMEE
        MEE101
            DOFMEE101
```

Additional Functions that are not called automatically:
MEEplotter

MAIN FUNCTION DESCRIPTIONS

humanMenu.m	The main MATLAB script. It calculates model results and outputs most of the results to a spreadsheet.
loadData.m	Loads the subject's data from spreadsheets into a structure array named "subject" in the MATLAB workspace.
**B_HSS.m**	Calculates the experimental BMR from the metabolic data
**E_HSS.m**	Calculates the experimental MEE from the metabolic data
**E_ACSM.m**	Estimates the MEE using ACSM formulas.
processSubject.m	Loads the subject specific data (mass, basal metabolic rate (BMR), etc.).
processStride.m	Loads the data for each trial/stride (joint torques, angular velocities, etc.).
calcResults.m	Calculates the model results and outputs them to spreadsheets.
**MEE.m**	Outputs the gross MEE, net MEE and the components of MEE (in Joules) of the whole body MEE predicted by the model for a given trial/stride.
**DOFMEE.m**	Similar to MEE.m, but outputs results for a given DOF instead of the whole

body.

**MEE101.m**	Outputs the instantaneous gross MEE, net MEE and the components of MEE (in Joules) for the whole body predicted by the model for a given trial/stride. The results are in a vector sampled from 0% to 100% of one gait cycle. In addition, it outputs the DS and SS related quantities such as total COT, SSCOT, DSCOT, etc.
**DOFMEE101.m**	Similar to MEE101, but outputs results for a given DOF instead of the whole body.
MEEplotter.m	Plots the model MEE, the components of model MEE, and the instantaneous over a gait cycle for a given trial/stride. It also outputs the results to a spreadsheet.

REFERENCES

[1] G. Wu, S. Siegler, P. Allard, C. Kirtley, A. Leardini, D. Rosenbaum, M. Whittle, D. D. D'Lima, L. Cristofolini, H. Witte, O. Schmid, and I. Stokes, "ISB recommendation on definitions of joint coordinate system of various joints for the reporting of human joint motion—Part I: ankle, hip, and spine," *J. Biomech.*, vol. 35, no. 4, pp. 543–548, Apr. 2002.

[2] G. Wu, F. C. T. van der Helm, H. E. J. (DirkJan) Veeger, M. Makhsous, P. Van Roy, C. Anglin, J. Nagels, A. R. Karduna, K. McQuade, X. Wang, F. W. Werner, and B. Buchholz, "ISB recommendation on definitions of joint coordinate systems of various joints for the reporting of human joint motion—Part II: shoulder, elbow, wrist and hand," *J. Biomech.*, vol. 38, no. 5, pp. 981–992, May 2005.

[3] M. A. Lafortune, P. R. Cavanagh, H. J. Sommer III, and A. Kalenak, "Three-dimensional kinematics of the human knee during walking," *J. Biomech.*, vol. 25, no. 4, pp. 347–357, Apr. 1992.
